# The Addition of EGFR Inhibitors in Neoadjuvant Therapy for KRAS-Wild Type Locally Advanced Rectal Cancer Patients: A Systematic Review and Meta-Analysis

**DOI:** 10.3389/fphar.2020.00706

**Published:** 2020-05-15

**Authors:** Xi Zhong, Yue Zhou, Wanbin Cui, Xin Su, Zhexu Guo, Iko Hidasa, Qincai Li, Zhenning Wang, Yongxi Song

**Affiliations:** ^1^Department of Surgical Oncology and General Surgery, Key Laboratory of Precision Diagnosis and Treatment of Gastrointestinal Tumors, Ministry of Education, The First Affiliated Hospital of China Medical University, Shenyang, China; ^2^Department of Medical Oncology, The First Affiliated Hospital of China Medical University, Shenyang, China; ^3^Department of Neurosurgery, The First Affiliated Hospital of China Medical University, Shenyang, China; ^4^Department of Urology, The Shengjing Hospital of China Medical University, Shenyang, China; ^5^Department of General Surgery, Yifeng Hospital of Traditional Chinese Medicine, Yichun, China

**Keywords:** locally advanced rectal cancer, kirsten rat sarcoma viral oncogene wild-type, neoadjuvant chemoradiotherapy, epidermal growth factor receptor inhibitors, cetuximab, panitumumab

## Abstract

**Background:**

Patients with locally advanced rectal cancer (LARC) are at higher risk of local and distant recurrence and are thus more vulnerable to metastatic diseases. Neoadjuvant chemoradiotherapy (nCRT) and subsequent curative resection with total mesorectal excision (TME) followed by adjuvant chemotherapy have been recommended by the National Comprehensive Cancer Network (NCCN) guidelines as standard of care for LARC patients. However, the efficacy of the addition of epidermal growth factor receptor (EGFR) inhibitors in kirsten rat sarcoma viral oncogene (KRAS)-wild type LARC patients remains uncertain.

**Materials:**

PubMed, Embase, and Web of Science were searched to retrieve records on the application of EGFR inhibitors in a neoadjuvant setting for LARC patients. pCR was used as surrogate endpoint to perform data synthesis in a single-arm setting.

**Results:**

Ten cohorts covering 540 subjects were eligible in this systematic review. The pooled pCR rate for EGFR inhibitors was 15% (95% confidence interval (95% CI), 11–20%; I^2^ = 55.2%); the pooled estimates of Grade 3/4 diarrhea, Grade 3/4 hand–foot syndrome, Grade 3/4 acneiform rash were 17% (95% CI, 4–34%; I^2^ = 93.3%), 2% (95% CI, 0–5%; I^2^ = 13.7%), and 15% (95% CI, 9–22%; I^2^ = 65.4%), respectively.

**Conclusion:**

The addition of EGFR inhibitors in the nCRT for KRAS-wild type LARC patients provides comparable efficacy and acceptable safety. However, the results should be interpreted cautiously due to the small amount of relevant data and need further confirmation by more future studies.

## Introduction

Rectal cancer has been recognized as one of the most prevalent and lethal cancers for decades (Torre et al., 2015). Patients with locally advanced rectal cancer (LARC) are at higher risk of local and distant recurrence and are thus more likely to suffer metastatic diseases (Schrag et al., 2014). To improve the local control and prognosis of LARC patients through tumor downstaging, the concept of neoadjuvant therapy, or performing preoperative chemoradiotherapy (CRT), has been introduced into clinical practice for years (Sauer et al., 2004; Bosset et al., 2006). According to the National Comprehensive Cancer Network (NCCN) guideline, neoadjuvant CRT (nCRT) and subsequent curative resection with total mesorectal excision (TME) followed by adjuvant chemotherapy have been recommended as standard of care for LARC patients (Rectal cancer V.2., 2020). However, considering the insufficient pathologic complete response (pCR) rates reported in clinical trials investigating various strategies in neoadjuvant setting, LARC patients are badly in need of nCRT regimens with better efficacy (Bosset et al., 2005; Gerard et al., 2006; Craven et al., 2007).

The application of targeted agents to improve the efficacy of nCRT in LARC patients has been a focus of research in the past decade. Our previous work (Zhong et al., 2018) comprehensively reviewed and evaluated the efficacy of the addition of antivascular endothelial growth factor (VEGF) or antiepidermal growth factor receptor (EGFR) targeted agents in meta-analyses of single-arm setting. We reported a promising pooled pCR rate of 27% in bevacizumab-relevant cohorts, but only that of 14% in cetuximab-relevant cohorts. Of note, the kirsten rat sarcoma viral oncogene (KRAS) mutation status of the cetuximab-relevant cohorts is not restricted to be wild-type due to the limitation of included studies, thus the insufficient pooled pCR rate may be accounted for by the potential KRAS-mutated subjects in these cohorts to some extent. In this regard, we performed this systematic review and meta-analysis to assess the addition of EGFR inhibitors in neoadjuvant therapy for KRAS-wild type LARC patients.

## Methods

### Study Selection

This systematic review was conducted according to the Preferred Reporting Items for Systematic Reviews and Meta-analysis (PRISMA) statements checklist (Moher et al., 2009).

The predefined criteria for inclusion and exclusion were: (1) Subjects diagnosed with LARC (clinical T stages 3–4 and/or lymph node metastasis, no distant metastatic diseases observed). (2) KRAS wild-type. (3) Administration of EGFR inhibitors in neoadjuvant setting. (4) Reported pCR and Grades 3–4 treatment-related toxicities such as diarrhea, hand–foot syndrome, and acneiform rash. (5) More recent and larger studies were chosen if research cohorts presented overlapping. (6) Original researches only, excluding reviews, systematic reviews, case reports, case series, letter to the editor.

### Search Strategy

PubMed, Embase, and Web of Science were searched for relevant publications from inception through August 28th, 2019 using the following terms: “rectal”, “rectum”, “colorectal”, “tumor”, “cancer”, “neoplasm”, “malignant”, “malignancy”, “malignancies”, “neoadjuvant”, “preoperative”, “perioperative”, “targeted”, “egfr”, “cetuximab”, “C225”, “panitumumab”, “nimotuzumab”. The search terms in details for each database are shown in **Supplementary Material S1**. References of the pertinent studies were manually screened for potential inclusion. No restriction of language or study design was used.

### Data Extraction

The endpoints of interest were pCR and the rates of patients who suffered Grade 3/4 treatment-related toxicities namely diarrhea, hand–foot syndrome, and acneiform rash. The following data concerning baseline characteristics of the eligible studies were extracted: first author and publication year, study design, country/district, subjects, strategy of neoadjuvant therapy, median age, and staging at enrollment. All data were independently extracted by two authors (Yue Zhou and Xi Zhong). Discrepancies were resolved through consensus. The methodological quality of the included studies was evaluated using the Newcastle–Ottawa quality assessment scale (NOS) (Stang, 2010). Studies which scored five or more were deemed of moderate-quality, whereas those with seven or more were considered high-quality (Stang, 2010).

### Statistical Analysis

Data of pCR and Grade 3/4 toxicity were within the range of 0–0.3, thus were firstly double arcsine transformed and then pooled using a random-effect model to provide more conservative estimates (Freeman and Tukey, 1950; Liebig et al., 2009). The Cochrane’s Q test and inconsistent index (I^2^) were performed to detect the presence of heterogeneity (Higgins et al., 2003). To detect potential origins of heterogeneity, subgroup analyses were performed based on the type of EGFR inhibitor, the intensity of backbone nCRT, and the region where each included study was conducted, which were selected following the PICOS (Participant, Intervention, Comparison, Outcome, and Study design) principle. A subgroup analysis based on study design was planned; however, considering that only one of the ten included studies was a retrospective study while the other nine were all prospective phase II studies, this subgroup analysis was not performed. Small study effects were evaluated using the Egger linear regression test when data was sufficient (≥10) (Sterne and Egger, 2001). All statistical analyses were performed using STATA version 12.0 (STATA, College Station, TX).

## Results

### Study Inclusion and the Baseline Characteristics of Eligible Studies

**Figure 1:**
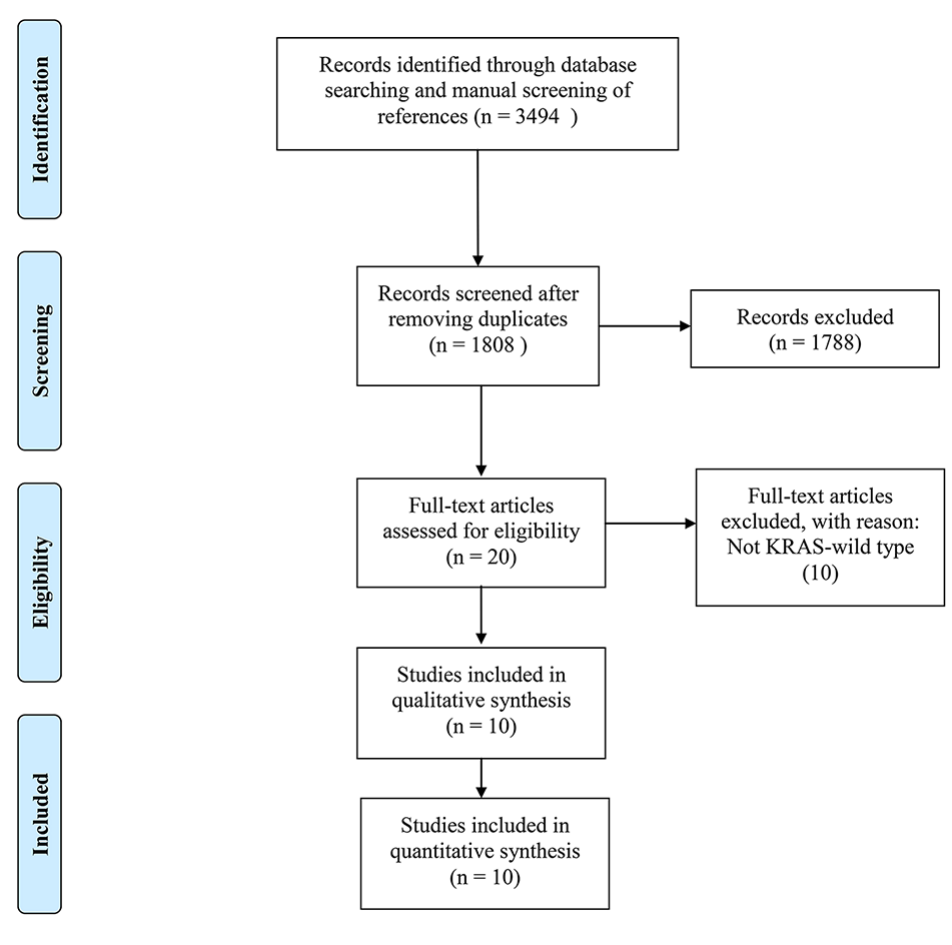
Literature search and study selection.

3,494 records were retrieved after database search and manual screening of references; 1,686 duplicates were removed leaving 1,808 records to proceed on title and abstract screening. 20 potential candidates underwent full-text review, of which 10 (Bengala et al., 2009; Pinto et al., 2011; Dewdney et al., 2012; Sun et al., 2012; Helbling et al., 2013; Liang et al., 2017; Hasegawa et al., 2017; Merx et al., 2017; Leichman et al., 2018; Pinto et al., 2018)were adequate for eligibility, after excluding 10 studies which were deemed inadequate because they did not focus on KRAS-wild type LARC patients (shown in **Figure 1**). Among the eligible studies, six (Bengala et al., 2009; Dewdney et al., 2012; Sun et al., 2012; Liang et al., 2017; Hasegawa et al., 2017; Leichman et al., 2018) focus on the efficacy of cetuximab while the other four (Pinto et al., 2011; Helbling et al., 2013; Merx et al., 2017; Pinto et al., 2018) panitumumab. Concerning the country/district where these trials were conducted, three (Bengala et al., 2009; Pinto et al., 2011; Pinto et al., 2018) were in Italy, one (Merx et al., 2017) in Germany, one (Helbling et al., 2013) in Switzerland & Hungary, one (Dewdney et al., 2012) in the United Kingdom, Spain & Sweden, one (Leichman et al., 2018) in the United States of America, one (Sun et al., 2012) in the mainland of China, one (Liang et al., 2017) in Taiwan, and one (Hasegawa et al., 2017) in Japan. The detailed baseline characteristics and data of the endpoints of interest of the included studies are shown in **Table 1**. The results of the methodological quality assessment of the eligible studies are shown in **Table 2**. Among the ten studies, two with seven points were deemed high-quality, while the remaining eight scored six points and were considered as moderate-quality studies.

**Table 1:** Baseline characteristics of cohort groups of EGFR inhibitors for meta-analysis.

Study	Study design	Country/District	Subjects	Neoadjuvant therapy	Median age, y	Stage at enrollment	Grade3/4 toxicity	pCR
Leichman et al., 2018	Prospective Phase II	USA	75	Induction therapy: Cetuximab + XELOX Concurrent chemoradiotherapy: Cetuximab + XELOX + RT	56.4	NR	Grade 3/4 diarrhea: 26/75 (34.7%) Grade 3/4 hand–foot syndrome: 2/75 (2.7%) Grade 3/4 acneiform rash: 9/75 (12%)	26.7% (20/75)
Pinto et al., 2018	Prospective Phase II	Italy	98	Panitumumab + RT	66.0	cT2N−: 2; cT2N+: 2; cT3N−: 33; cT3N+: 56	Grade 3/4 diarrhea: 2/98 (2.0%) Grade 3/4 hand–foot syndrome: NR Grade 3/4 acneiform rash: 16/98 (16.3%)	10.9% (10/92)
Merx et al., 2017	Prospective Phase II	Germany	59	Panitumumab + RT	58.4	cT2: 4; cT3: 50; cN+: 49; cN−: 5	Grade 3/4 diarrhea: 6/59 (10.2%) Grade 3/4 hand–foot syndrome: NR Grade 3/4 acneiform rash: 14/59 (23.7%)	3.7% (2/54)
Hasegawa et al., 2017	Prospective Phase II	Japan	40	Cetuximab + mFOLFOX6	64.5	cT2: 1; cT3: 34; cT4a: 5; cN0: 22; cN1-2: 18	NR	17.5% (7/40)
Liang et al., 2017	Retrospective	Taiwan	48	Cetuximab + FOLFOX	59.0	cT3N0: 29; cT3N1: 14; cT3N2: 5	NR	20.8% (10/48)
Helbling et al., 2013	Prospective Phase II	Switzerland & Hungary	40	Panitumumab + Capecitabine + RT	62.0	cT2: 4; cT3: 34; cT4: 2; cN0: 8; cN1: 24; cN2: 8	Grade 3/4 diarrhea: 4/40 (10%) Grade 3/4 hand–foot syndrome: 1/40 (2.5%) Grade 3/4 acneiform rash: 1/40 (2.5%)	10% (4/40)
Dewdney et al., 2012	Prospective Phase II	UK, Spain & Sweden	46	Cetuximab + Capecitabine + RT	59.0	cT3c- T3d: 23; T4: 12	NR	10.9% (5/46)
Sun et al., 2012	Prospective Phase II	China (mainland)	44	Cetuximab + Capecitabine + RT	NR	NR	NR	13.6% (6/44)
Pinto et al., 2011	Prospective Phase II	Italy	60	Panitumumab + 5-FU + oxaliplatin + RT	60.0	cT3N+: 41; cT4N−: 4; cT4N+: 11; cT3Nx: 1; cT4Nx: 3	Grade 3/4 diarrhea: 23/60 (38.33%) Grade 3/4 hand–foot syndrome: 0 Grade 3/4 acneiform rash: 11/60 (18.33%)	21.1% (12/57)
Bengala 2009	Prospective Phase II	Italy	30	Cetuximab + 5-FU+RT	NR	NR	NR	10% (3/30)

pCR, pathologic complete response; RT, radiotherapy; 5-FU, fluorouracil; FOLFOX, leucovorin plus fluorouracil plus oxaliplatin; XELOX, capecitabine plus oxaliplatin; y, year; NR, not reported.

**Table 2:** The NOS quality of included studies.

Study	Selection				Comparability		Outcome			Total	Quality
	REC	SNEC	AE	DO	SC	AF	AO	FU	AFU		
Leichman et al., 2018	1	0	1	1	0	0	1	1	1	6	Moderate
Pinto et al., 2018	1	0	1	1	0	0	1	1	1	6	Moderate
Merx et al., 2017	1	0	1	1	0	0	1	1	1	6	Moderate
Hasegawa et al., 2017	1	0	1	1	0	0	1	1	1	6	Moderate
Liang et al., 2017	1	0	1	1	0	0	1	1	1	6	Moderate
Helbling et al., 2013	1	1	1	1	0	0	1	1	1	7	Moderate
Dewdney et al., 2012	1	1	1	1	0	0	1	1	1	7	High
Sun et al., 2012	1	0	1	1	0	0	1	1	1	6	Moderate
Pinto et al., 2011	1	0	1	1	0	0	1	1	1	6	Moderate
Bengala et al., 2009	1	0	1	1	0	0	1	1	1	6	Moderate

REC, representativeness of the exposed cohort; SNEC, selection of the nonexposed cohort; AE, ascertainment of exposure; DO, demonstration that outcome of interest was not present at start of study; SC, study controls for age, sex; AF, study controls for any additional factors; AO, assessment of outcome; FU, follow-up long enough for outcomes to occur; AFU, adequacy of follow-up of cohorts (≥90%). “1” means that the study satisfied the item and “0” means the opposite situation.

### The Efficacy of EGFR Inhibitors

**Figure 2:**
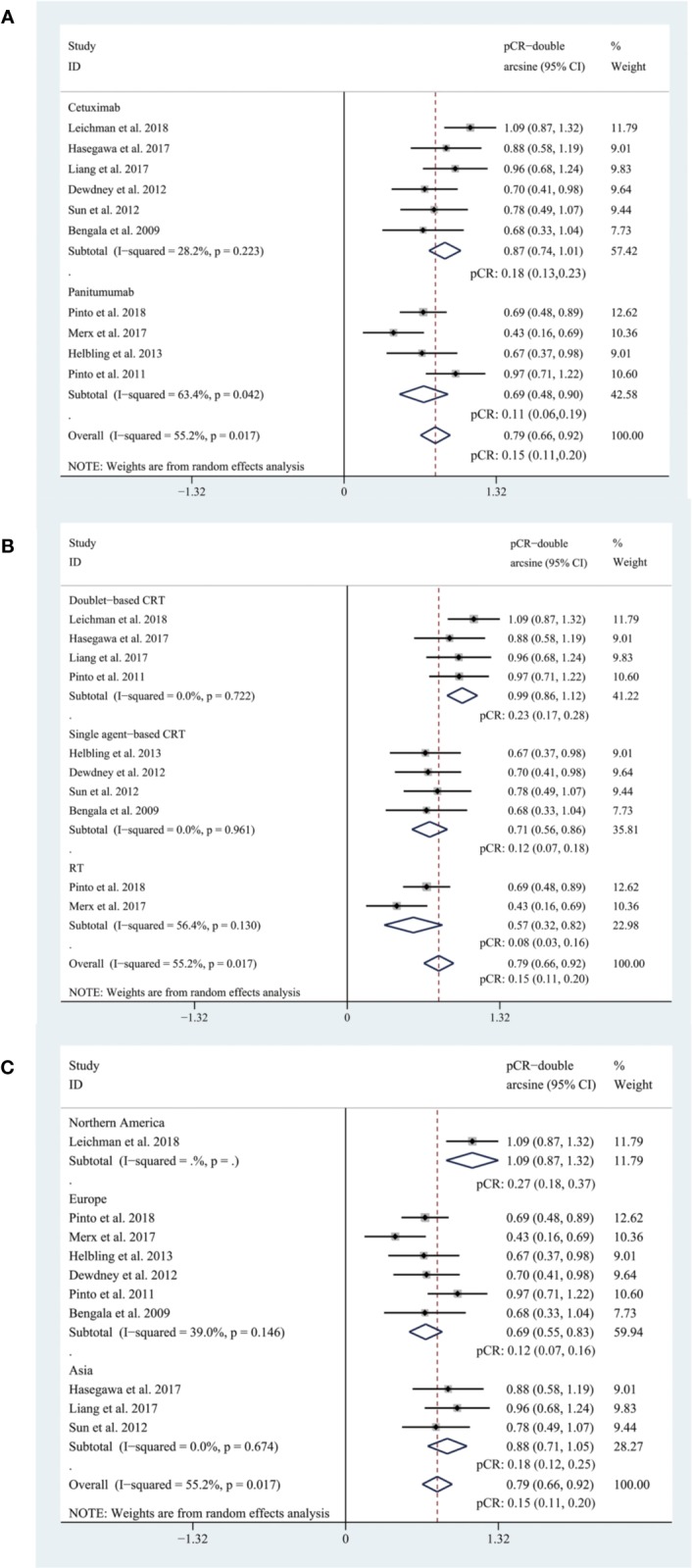
**(A)** The forest plot of pooled estimate of pCR (subgrouped by the type of EGFR inhibitor); **(B)** the forest plot of pooled estimate of pCR (subgrouped by the intensity of backbone nCRT); **(C)** the forest plot of pooled estimate of pCR (subgrouped by region).

**Figure 3:**
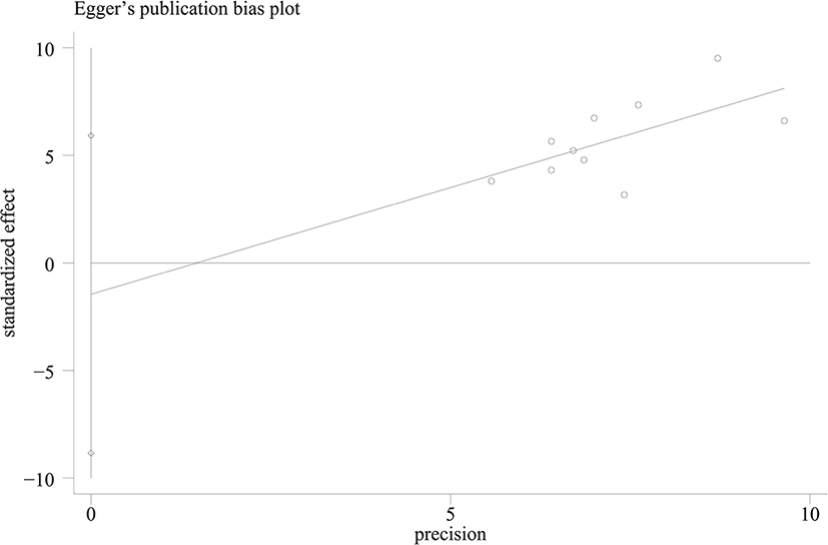
The Egger’s funnel plot of pooled pCR.

The pooled pCR rate for EGFR inhibitors was 15% (95% confidence interval (95% CI), 11–20%; I^2^ = 55.2%) as shown in **Figure 2**. According to the results of subgroup analyses based on the type of EGFR inhibitor, the intensity of backbone nCRT and the region where each included study was conducted, the pooled pCR rate for cetuximab-based cohorts was 18% (95% CI, 13–23%; I^2^ = 28.2%) while that for panitumumab-based cohorts was 11% (95% CI, 6–19%; I^2^ = 63.4%) (**Figure 2**); the pooled pCR rates for doublet-based CRT-, single agent-based CRT-, and RT-relevant cohorts were 23% (95% CI, 17–28%; I^2^ = 0%), 12% (95% CI, 7–18%; I^2^ = 0%), and 8% (95% CI, 3–16%; I^2^ = 56.4%), respectively (**Figure 2**); the pooled pCR rates for North America-, Europe-, and Asia-originated cohorts were 27% (95% CI, 18–37%, I^2^ not available), 12% (95% CI, 7–16%; I^2^ = 39.0%), and 18% (95% CI, 12–25%; I^2^ = 0%), respectively (**Figure 2**). No small study effects were detected as the *P* value of Egger’s test was 0.660 (**Figure 3**).

### The Safety of EGFR Inhibitors

**Figure 4:**
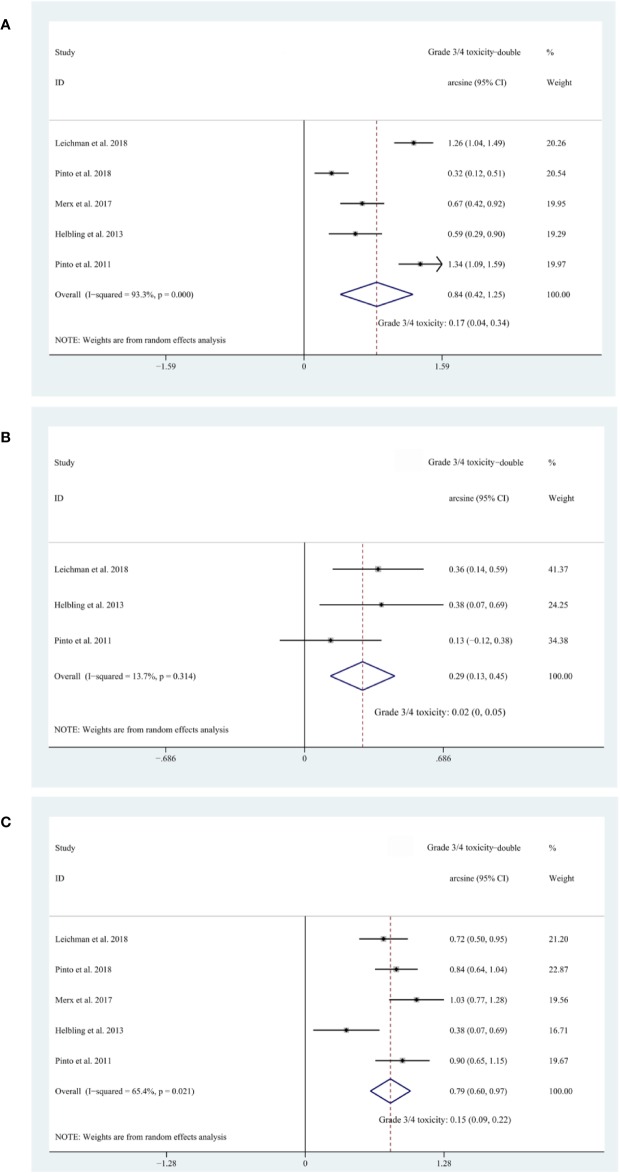
**(A)** The forest plot of pooled estimate of Grade 3/4 diarrhea; **(B)** the forest plot of pooled estimate of Grade 3/4 hand–foot syndrome; **(C)** the forest plot of pooled estimate of Grade 3/4 acneiform rash.

Five cohorts (Pinto et al., 2011; Helbling et al., 2013; Merx et al., 2017; Leichman et al., 2018; Pinto et al., 2018) reported on Grade 3/4 diarrhea, three (Pinto et al., 2011; Helbling et al., 2013; Leichman et al., 2018) reported on Grade 3/4 hand–foot syndrome, and five (Pinto et al., 2011; Helbling et al., 2013; Merx et al., 2017; Leichman et al., 2018; Pinto et al., 2018) reported on Grade 3/4 acneiform rash. The pooled estimates of Grade 3/4 diarrhea, Grade 3/4 hand–foot syndrome, Grade 3/4 acneiform rash were 17% (95% CI, 4–34%; I^2^ = 93.3%), 2% (95% CI, 0–5%; I^2^ = 13.7%), and 15% (95% CI, 9–22%; I^2^ = 65.4%), respectively (**Figure 4**). Subgroup analyses and Egger’s test were not performed due to the insufficient amount of data.

## Discussion

### Main Findings and Interpretation in Light of the Evidence

KRAS mutation was firstly demonstrated as predictive for lack of response in 2008 (Amado et al., 2008; Karapetis et al., 2008); the studies investigating the roles of EGFR inhibitors in the nCRT for KRAS-wild type LARC patients arose ever since. However, these studies are mostly signal-seeking single-arm phase II trials using pCR, a well-established surrogate endpoint for survival outcomes, as primary endpoint, largely lacking head-to-head survival data comparing neoadjuvant regimens with or without anti-EGFR targeted agents (Bengala et al., 2009; Pinto et al., 2011; Helbling et al., 2013; Zhong et al., 2018). In 2014, an important phase II randomized controlled trial (RCT) (EXPERT-C) by Dewdney et al. (2012). reported a significant improvement in overall survival for KRAS wild-type LARC patients receiving neoadjuvant XELOX and cetuximab (hazard ratio, 0.27; 95% CI, 0.07–0.99; P = 0.034). However, the primary endpoint, pCR, was only 11% in the cetuximab arm compared with 7% in the control arm. In another RCT (SAKK 41/07), a pCR of 10% was reached in KRAS wild-type LARC patients treated with panitumumab and capecitabine compared with 18% in those treated with capecitabine. Of note, the cetuximab/panitumumab arms in these RCTs were included in this systematic review and meta-analysis, while the other eight included studies are either single-arm phase II clinical trials (Bengala et al., 2009; Pinto et al., 2011; Dewdney et al., 2012; Sun et al., 2012; Hasegawa et al., 2017; Merx et al., 2017; Leichman et al., 2018; Pinto et al., 2018) (7) or single arm retrospective study (Liang et al., 2017) (1). On account of the single arm nature of the ten included cohorts and the widely-acknowledged prognostic role of pCR, we decided to conduct a single arm meta-analysis using pCR as primary endpoint to evaluate the efficacy of neoadjuvant EGFR inhibitors in LARC patients. Besides, to provide a benchmark pCR rate, in our previous work (Zhong et al., 2018), we extracted and pooled relevant data from ten LARC cohorts extracted from the individual patient data-leveled meta-analysis of Maas et al (Maas et al., 2010. In this work, we transformed these data using double arcsine and synthesized a pooled pCR rate of 15% (95% CI, 13–17%), as shown in **Figure S1**, compared with that of 17% (95% CI, 15–20%) in the previous work where we did not use double arcsine transformation. The baseline characteristics of included cohorts are presented in **Supplementary Table S1**.

In this systematic review and meta-analysis, we reached a pooled pCR rate of 15% (95% CI, 11–20%) for all EGFR-inhibitor-relevant cohorts, which is comparable to the new benchmark set at 15%. According to subgroup analyses, the pooled pCR rate for cetuximab-relevant cohorts (18%, 95% CI: 13–23%, I^2^ = 28.2%) is higher than that for panitumumab-relevant cohorts (11%, 95% CI: 6–19%, I^2^ = 63.4%). The insufficient efficacy of neoadjuvant panitumumab for LARC patients and the higher heterogeneity may be accounted for by the backbone nCRT of these cohorts. Two (Merx et al., 2017; Pinto et al., 2018) of total four panitumumab-relevant cohorts received panitumumab plus concurrent radiotherapy (RT), the other two received panitumumab, capecitabine or 5-FU plus RT, with (Pinto et al., 2011) or without (Helbling et al., 2013)). oxaliplatin while cetuximab-relevant cohorts received more intense backbone nCRT regimen. To further account for the intensity of backbone nCRT, a subgroup analysis was performed and the pooled pCR rates for doublet-based CRT-, single agent-based CRT-, and RT-relevant cohorts were 23% (95% CI, 17–28%; I^2^ = 0%), 12% (95% CI, 7–18%; I^2^ = 0%), and 8% (95% CI, 3–16%; I^2^ = 56.4%), respectively. Based on the results, it’s reasonable to come to a conclusion that cohorts treated with more intensified backbone nCRT reach higher pCR rates and that the heterogeneity basically originates from RT-relevant cohorts (I^2^ = 56.4%) rather than the other two subgroups (I^2^ = 0% for both). Moreover, the pooled pCR rate for Europe-originated cohorts (12%, 95% CI: 7–16%, I^2^ = 39.0%) is inferior to that for Asia- (18%, 95% CI: 12–25%, I^2^ = 0%) and North America-originated cohorts (27%, 95% CI: 18–37%, I^2^ not available), which may also be explained by the intensity of backbone nCRT. Of note, the North America-originated cohort (Leichman et al., 2018) evaluated the efficacy of the addition of cetuximab in oxaliplatin-based induction therapy and concurrent nCRT and reached the highest pCR rate among all included cohorts.

To evaluate the safety of additional EGFR inhibitors, we synthesized the risk of Grade 3/4 treatment-related toxicities namely Grade 3/4 diarrhea (17%, 95% CI: 4–34%), Grade 3/4 hand–foot syndrome (2%, 95% CI: 0–5%), and Grade 3/4 acneiform rash (15%, 95% CI: 9–22%). This safety is acceptable considering that previously published clinical trials reported overall risks of Grade 3/4 toxicity ranging from 13.9% to 27% (Sauer et al., 2004; Bosset et al., 2006; Gerard et al., 2006; Rodel et al., 2012). However, the intensity of backbone nCRT regimens of the included cohorts in safety evaluation is weaker than that of these previous studies, thus more future studies on more intense backbone nCRT regimens are needed to level this intensity gap.

#### Strengths and Limitations

This is the most comprehensive systematic review to evaluate the efficacy and safety of the addition of EGFR inhibitors in the nCRT for KRAS-wild type LARC patients in a single-arm setting, and we used double arcsine transformation to process data to better cope with the feature of all these data lying within the range of 0 to 0.3.

Nonetheless, there are several limitations that have to be addressed. First, lack of head-to-head survival data weakens our analyses; pCR is only a surrogate endpoint for prognosis. Second, more future studies evaluating the addition of EGFR inhibitors in neoadjuvant therapy for KRAS-wild type LARC patients are needed to provide more stable and more robust results. Third, due to the lack of currently available studies, this study focused solely on KRAS status; however, increasing evidences demonstrate that the response to anti-EGFR treatment can be also strictly correlated with the mutational statuses of NRAS and BRAF (Sorich et al., 2015; Sanz-Garcia et al., 2017). Future studies stressing more on NRAS and BRAF statuses are warranted. Fourth, although the significant heterogeneity appears to originate from RT-relevant cohorts, only two studies were included in this subgroup. The intensity of backbone nCRT requires to be further accounted for as the amount of eligible trials increases in the future. The preferred backbone nCRT is 5-FU/capecitabine with concurrent RT, considering that the addition of oxaliplatin is now not recommended in neoadjuvant setting for LARC patients, according to the latest version of NCCN guidelines (Rectal cancer V.2., 2020).

## Conclusion

The addition of EGFR inhibitors in the nCRT for KRAS-wild type LARC patients provides comparable efficacy and acceptable safety. However, the results should be interpreted cautiously due to the small amount of relevant data and need further confirmation by more future studies.

## Data Availability Statement

The raw data supporting the conclusions of this article will be made available by the authors, without undue reservation, to any qualified researcher.

## Author Contributions

XZ, YZ, and YS conceived and designed the study. XZ and YZ performed the literature search, acquired and collated the data, which were analyzed by XZ, WC, XS, ZG, IH, QL, and ZW. All authors drafted and critically revised the manuscript for important intellectual content. All authors gave final approval of the version to be published and contributed to the manuscript. YS and ZW are guarantors. The corresponding authors attest that all listed authors meet authorship criteria and that no others meeting the criteria have been omitted.

## Funding

This work was supported by the Natural Science Foundation of Liaoning Province (No. 20180550582), Xingliao Talents Program in Liaoning Province (XLYC1807164), Project of Science and Technology of Shenyang (No. 18-014-4-07), and China Postdoctoral Science Foundation (No. 2018M640267). The funders did not have any influence on the study design; in the collection, analysis, and interpretation of data; in the writing of the report; and in the decision to submit the article for publication.

## Conflict of Interest

The authors declare that the research was conducted in the absence of any commercial or financial relationships that could be construed as a potential conflict of interest.

## Supplementary Material

Figure S1 The forest plot of pooled estimate of pCR using data extracted from the individual participant data-based pooled analysis by Maas et al.
