# Supplementary figures and images for: The Addition of EGFR Inhibitors in Neoadjuvant Therapy for KRAS-Wild Type Locally Advanced Rectal Cancer Patients: A Systematic Review and Meta-Analysis
Source: Front Pharmacol. 2020 May 15;11:706. doi: 10.3389/fphar.2020.00706 (PMC7242658; doi:10.3389/fphar.2020.00706)

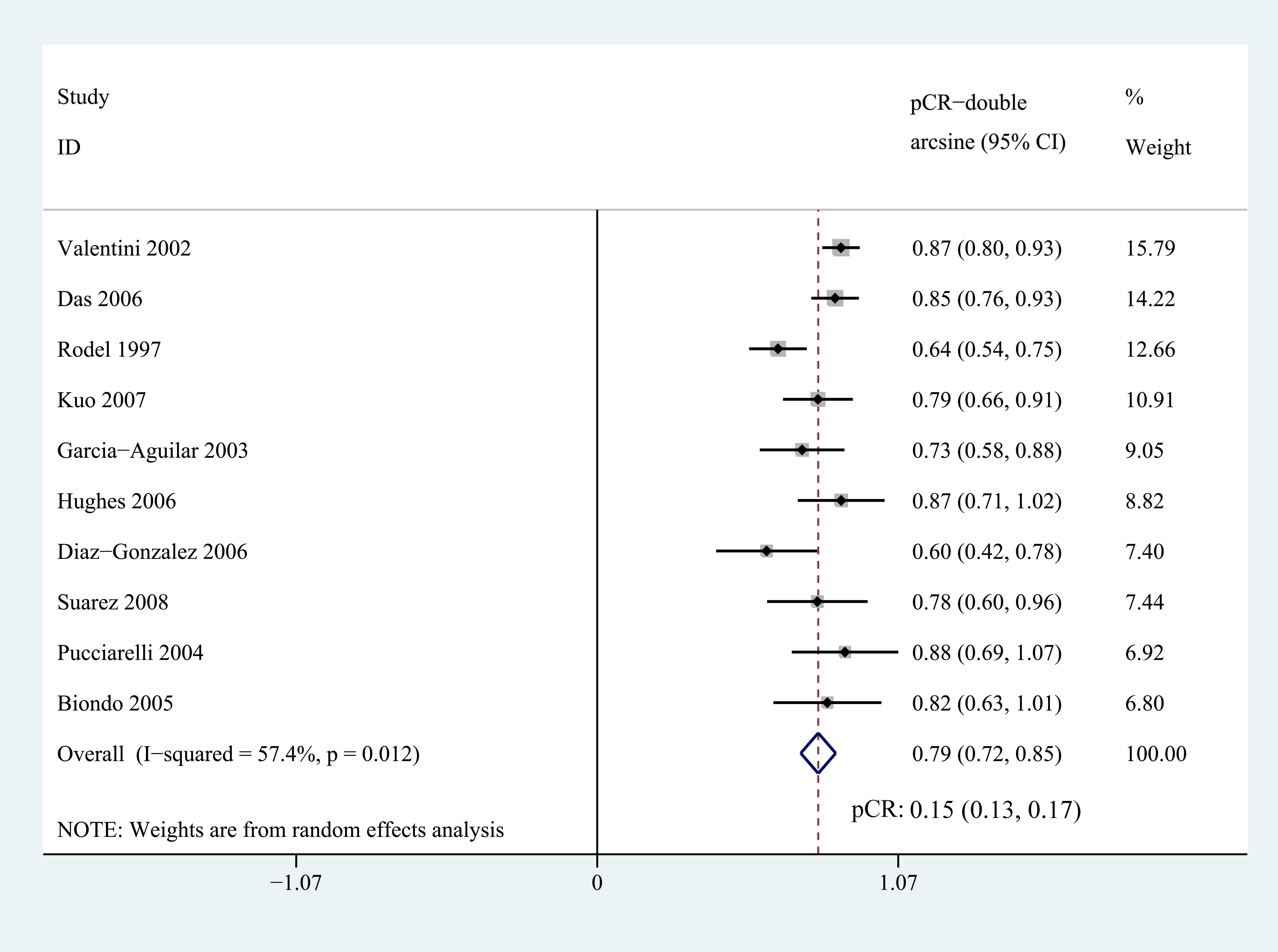

Supplement: Figure S1 — The forest plot of pooled estimate of pCR using data extracted from the individual participant data-based pooled analysis by Maas et al. [file Image_1.jpeg]
