# Supplementary material for: The Addition of EGFR Inhibitors in Neoadjuvant Therapy for KRAS-Wild Type Locally Advanced Rectal Cancer Patients: A Systematic Review and Meta-Analysis
Source: Front Pharmacol. 2020 May 15;11:706. doi: 10.3389/fphar.2020.00706 (PMC7242658; doi:10.3389/fphar.2020.00706)
Supplement: Supplementary file 3 [file DataSheet_1.docx]

Search details

PubMed:

(“rectal”[All Fields] OR "rectum"[MeSH Terms] OR colorectal[All Fields]) AND ("tumor"[All Fields] OR “cancer”[All Fields] OR "neoplasms"[MeSH Terms] OR “malignant”[All Fields] OR “malignancy”[All Fields] OR “malignancies”[All Fields]) AND (“neoadjuvant” [All Fields] OR “preoperative” [All Fields] OR “perioperative” [All Fields]) AND (targeted[All Fields] OR "egfr"[All Fields] OR "cetuximab"[MeSH Terms] OR "c225"[All Fields] OR "panitumumab"[MeSH Terms] OR "nimotuzumab"[All Fields])

Embase:

(rectal OR 'rectum'/exp OR rectum OR colorectal) AND ('tumor'/exp OR tumor OR 'cancer'/exp OR cancer OR 'neoplasm'/exp OR neoplasm OR malignant OR 'malignancy'/exp OR malignancy OR 'malignancies'/exp OR malignancies) AND (neoadjuvant OR preoperative OR perioperative) AND (targeted OR 'egfr'/exp OR egfr OR 'cetuximab'/exp OR cetuximab OR 'c225'/exp OR c225 OR 'panitumumab'/exp OR panitumumab OR 'nimotuzumab'/exp OR nimotuzumab)

Web of Science:

TOPIC: (((((rectal OR rectum) OR colorectal) AND (((((tumor OR cancer) OR neoplasm) OR malignant) OR malignancy) OR malignancies)) AND ((neoadjuvant OR preoperative) OR perioperative)) AND (((((targeted OR egfr) OR cetuximab) OR c2h5) OR panitumumab) OR nimotuzumab))
